# T cells enhance gold nanoparticle delivery to tumors *in vivo*

**DOI:** 10.1186/1556-276X-6-283

**Published:** 2011-04-04

**Authors:** Laura C Kennedy, Adham S Bear, Joseph K Young, Nastassja A Lewinski, Jean Kim, Aaron E Foster, Rebekah A Drezek

**Affiliations:** 1 Department of Bioengineering, Rice University, Houston, TX 77005, USA; 2 Center for Cell and Gene Therapy, Baylor College of Medicine, The Methodist Hospital and Texas Children's Hospital, Houston, TX 77030, USA; 3 Department of Electrical and Computer Engineering, Rice University, Houston, TX 77005, USA

## Abstract

Gold nanoparticle-mediated photothermal therapy (PTT) has shown great potential for the treatment of cancer in mouse studies and is now being evaluated in clinical trials. For this therapy, gold nanoparticles (AuNPs) are injected intravenously and are allowed to accumulate within the tumor via the enhanced permeability and retention (EPR) effect. The tumor is then irradiated with a near infrared laser, whose energy is absorbed by the AuNPs and translated into heat. While reliance on the EPR effect for tumor targeting has proven adequate for vascularized tumors in small animal models, the efficiency and specificity of tumor delivery *in vivo*, particularly in tumors with poor blood supply, has proven challenging. In this study, we examine whether human T cells can be used as cellular delivery vehicles for AuNP transport into tumors. We first demonstrate that T cells can be efficiently loaded with 45 nm gold colloid nanoparticles without affecting viability or function (e.g. migration and cytokine production). Using a human tumor xenograft mouse model, we next demonstrate that AuNP-loaded T cells retain their capacity to migrate to tumor sites *in vivo*. In addition, the efficiency of AuNP delivery to tumors *in vivo *is increased by more than four-fold compared to injection of free PEGylated AuNPs and the use of the T cell delivery system also dramatically alters the overall nanoparticle biodistribution. Thus, the use of T cell chaperones for AuNP delivery could enhance the efficacy of nanoparticle-based therapies and imaging applications by increasing AuNP tumor accumulation.

## Introduction

Gold nanoparticles (AuNPs) have been successfully used to enable photothermal therapy (PTT) for the treatment of cancer in small animal studies [1-5], and has recently moved towards clinical application [6]. A variety of AuNPs have been examined for PTT, including silica-gold nanoshells [2], gold nanorods [7], gold nanocages [8], gold-gold sulfide nanoparticles [9], and hollow gold nanoshells [3]. These particles can be engineered to absorb light in the near infrared (NIR) range, where light is maximally transmissive and minimally absorbed by tissue. AuNP delivery can be accomplished by systemic administration (intravenous injection). The nanoparticles, depending on their shape, hydrodynamic size, and surface charge, will accumulate within the tumor via its irregular vasculature; this passive accumulation is known as the enhanced permeability and retention (EPR) effect [10,11]. Polyethylene glycol (PEG)-coated particles with a hydrodynamic diameter of approximately 60 nm have demonstrated the most efficient delivery to the tumor using the EPR effect [11], and more recently, the use of smaller hollow gold [3] and gold-gold sulfide nanoshells [12] (20-40 nm diameter) has further improved nanoparticle half-life in the blood. However, even with these smaller particles, the percentage of the injected dose (ID) delivered to the tumor is low [13,14], and accumulation in non-target sites such as the liver and spleen is comparatively high [13,15]. Thus, new methods aimed at improving tumor delivery and specificity may increase the tumor concentration of gold nanoparticles and ultimately the efficacy of anti-tumor PTT and enhance nanoparticle mediated imaging techniques.

Attempts at augmenting gold nanoparticle tumor delivery have included a variety of nanoparticle surface modifications, including conjugation with antibodies [3] and hormone analogs [16]; however, inclusion of targeting ligands has only modestly improved tumor accumulation and specificity. This is likely due to the reliance of these nanoparticles on passive accumulation through EPR, which is highly dependent on adequate blood flow to the tumor. Therefore, tumors or tumor regions that exhibit poor vasculature and hypoxia are less likely to be effectively targeted using nanoparticles, ultimately limiting their therapeutic use. Choi et al. [17] recently demonstrated that macrophages could be used as a cellular delivery vehicle to deposit AuNPs in tumors and hypoxic tumor tissues, facilitating delivery through active cellular migration and extravasation in response to chemotactic factors produced by malignant cells. These encouraging results suggested that other immune cells might be used as cellular delivery vehicles. In this study, we assessed the capacity of activated T cells to function as chaperones for AuNPs. Unlike macrophage, T cells are readily isolated and expanded *in vitro*, and upon infusion, circulate throughout the body and migrate into tumors in response to tumor-associated chemokines. This tumor-tropic property permits their use as cellular vehicles for the delivery of molecular therapeutics [18-21].

Combining the advantages of T cells with nanotechnology has the potential to generate innovative new approaches to cancer therapy. Several studies have demonstrated that T cells may serve as efficient drug delivery vehicles for the treatment of cancer, including transport of magnetic particles bearing doxorubicin and for use in boron neutron capture therapy [22,23]. Here we have explored whether T cells can be used as AuNP carriers to increase delivery to tumor sites *in vivo *using gold colloidal nanospheres (40-45 nm), comparable in size to hollow gold nanoshells and gold-gold sulfide nanoparticles used for PTT. Although gold colloid in this size range has maximal absorbance in the visible wavelengths, there are several variants of AuNPs that are of similar size (25-60 nm) and absorb optimally in the NIR region, permitting translation of this delivery method for PTT. These gold nanoparticle variants include gold-gold sulfide nanoparticles, hollow gold nanoshells, and gold nanocubes, all of which have demonstrated efficacy as PTT-mediating agents in mouse studies [24]. Additionally, there have been studies demonstrating photothermal therapy using gold colloid that has been strategically aggregated to red-shift the peak absorbance into the NIR [25,26]. The possible applications of a AuNP-T cell delivery system could further be extended to imaging and drug delivery applications, as gold nanoparticles also have demonstrated potential as scatter- and absorption-based imaging contrast agents [27-30] and drug delivery agents [31]. In this study, we demonstrate that gold colloid is readily taken up by activated human T cells without impairing their viability or cellular functions, and that following intravenous infusion into tumor bearing micecan more efficiently deliver AuNPs to distant tumor sites.

## Results

### Loading of activated human T cells with AuNPs

**Figure 1:**
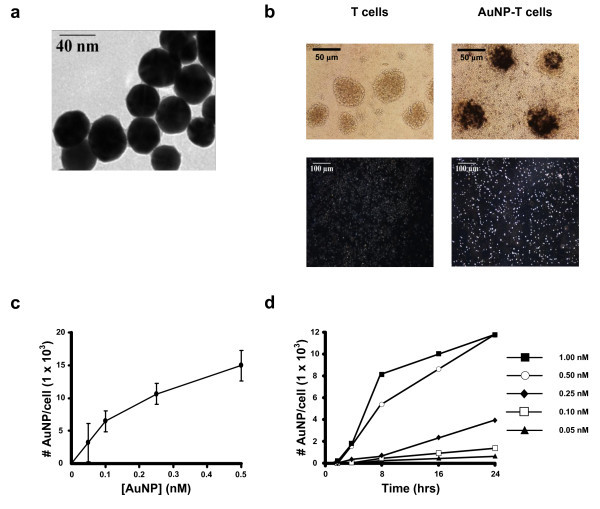
**Gold nanoparticle uptake by human T cells**. **(A) **TEM imaging of gold colloid (diameter = 40-45 nm). **(B) **Brightfield (upper) and darkfield (lower) images of human T cells demonstrate gold nanoparticle uptake by the increased light scattering seen in the AuNP-T cell group compared to T cells alone. **(C) **ICP-OES analysis of T cell gold content at 24 h using different nanoparticle loading concentrations. Each point is a composite of data acquired from three different T cell donors. **(D) **Time course data for T cells from a single donor loaded with different concentrations of gold nanoparticles. Optimal loading occurred after 24 h at a concentration between 0.5 and 1.0 nM.

Synthesized gold colloid was determined to be 40-45 nm in diameter by transmission electron microscopy (TEM) (Figure 1a and Figure S1 in Additional file 1). Activated and expanded human T cells were cultured in the presence of AuNPs for a period of 1 to 24 h to permit AuNP internalization. AuNP loading was confirmed using bright field and dark field microscopy demonstrating that T cells co-localize with AuNPs (Figure 1b). We further optimized loading conditions by altering AuNP concentration (per cell) and time of incubation. To determine the number of nanoparticles present per T cell, an inductively coupled plasma optical emission spectrometry (ICP-OES) analysis was used. T cells from three different human donors were first cultured with concentrations of AuNPs ranging from 0.05 to 0.5 nM for a period of 24 h to evaluate for variability in gold nanoparticle loading due to differences in T cells from different donors (Figure 1c). A maximum of 14,900 ± 2,400 AuNPs was internalized per T cell using a AuNP loading concentration of 0.5 nM (Figure 1c). We then performed a time course study using T cells from a single donor to determine the minimum amount of time required to load the T cells with the maximum number of AuNPs (Figure 1d). For this study, we incubated T cells with nanoparticle concentrations ranging from 0.05 to 1 nM. At 24 h, the 0.5 and 1 nM groups have similar gold content, suggesting that there is a maximum amount of AuNPs that can be internalized by T cells. These results demonstrate that maximal AuNP loading of T cells can be achieved using a concentration of 0.5 nM AuNP and an incubation period of 24 h.

### AuNP-loading does not affect T cell viability or function

**Figure 2:**
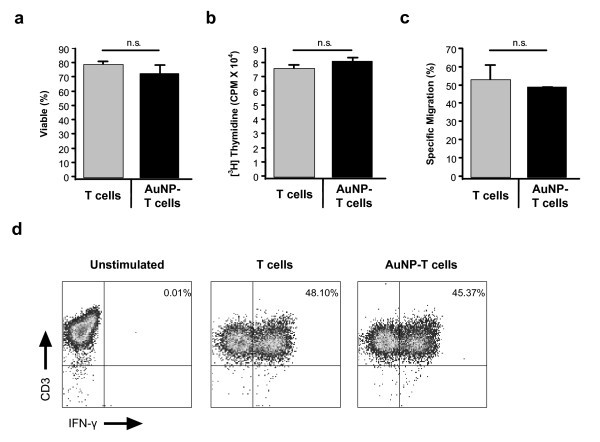
**AuNP loading has no significant effect on T cell viability or function**. T cells were loaded in the presence of 0.5 nM AuNPs for 24 h or cultured in medium alone and then measured for viability using Annexin-V/PI staining **(A)**, proliferation using thymidine incorporation **(B)**, migration through a transwell membrane in response to tumor (LCL) produced supernatant **(C) **and intracellular analysis of IFN-γ cytokine production following mitogen stimulation **(D)**.

We next measured T cell viability and function post-AuNP loading to assess potential toxicity that may inhibit T cell performance as an *in vivo *delivery vehicle. Loading T cells with AuNPs had no immediate effect on T cell viability as determined by Annexin-V/7-AAD staining (Figure 2a) and did not alter the phenotype of the cells (Figure S2 in Additional file 1). Furthermore, there were no prolonged effects on T cell proliferation as measured by thymidine incorporation (Figure 2b). Importantly, AuNPs did not affect migration when tested in a transwell chemotaxis assay against supernatant produced from human LCL tumors, suggesting that T cells retain their migratory behavior post-AuNP loading (Figure 2c). Finally, production of IFN-γ following mitogen activation (PMA-I) was not impaired by AuNPs (Figure 2d). These results show that AuNPs have no detrimental effects on T cell viability and function *in vitro *and indicate that T cell migration *in vivo *will likely be retained following loading.

### T cells migrate and transport AuNPs to tumors *in vivo*

**Figure 3:**
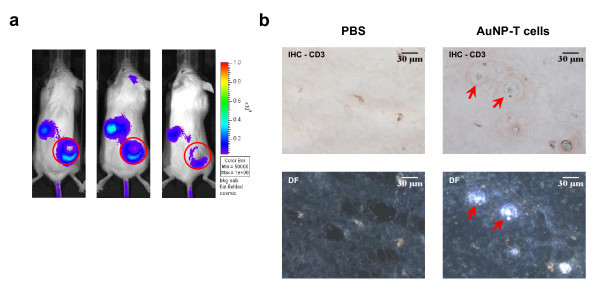
**AuNP-loaded T cells migrate to tumors *in vivo***. **(A) **T cells were retrovirally modified to express firefly luciferase then loaded in the presence of 0.5 nM AuNP for 24 h. Cells were subsequently injected intravenously into SCID mice bearing subcutaneous xenografted LCL tumors. Bioluminescent imaging of AuNP-T cell biodistribution at 48 h post-injection showing AuNP-T cell localization at the tumor site (red circle) and within the spleen. **(B) **Resected tumors were analyzed by bright field imaging (top row) and immunohistochemistry for human CD3 expression and dark field imaging (bottom row) to indicate the presence of AuNPs. Red arrows indicate the colocalization of CD3^+ ^T cells and AuNPs within the tumor.

*In vivo *AuNP-T cell migration to tumor sites was first examined using bioluminescent imaging and histology. T cells were first genetically modified to express firefly luciferase and then subsequently loaded with AuNPs. Bioluminescent imaging 48 h post-intravenous injection of AuNP-T cells demonstrate specific migration of the T cells to subcutaneous LCL tumors in immune deficient SCID mice (Figure 3a). This timepoint was selected based on previous studies that have demonstrated T cell localization to tumor sites 48 h post-infusion [32,33]. We next resected the tumors and performed histology to determine if AuNPs and T cells co-localized within the tumor. Immunohistochemical staining using CD3 antibody (a pan-T cell marker) demonstrated infiltration of T cells into the tumor (Figure 3b). In addition, areas of increased scatter in the darkfield images correlated well with areas of CD3^+ ^staining. This observation demonstrates that the T cells maintain internalized AuNPs during *in vivo *migration to the tumor site.

### Delivery of AuNPs by T cells alters nanoparticle biodistribution

**Figure 4:**
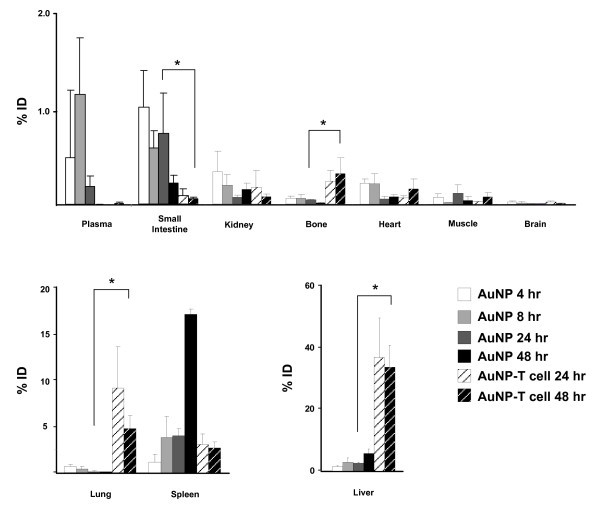
**Biodistribution comparison of AuNPs and AuNP-T cells in mice**. Mice were injected with PEG-AuNPs (60-65 nm hydrodynamic diameter), AuNP-T cells, or PBS and subsequently sacrificed at various time points to determine biodistribution. PBS gold levels were negligible in comparison to AuNP and AuNP-T cell groups for all organs. Values are percentage of the injected gold dose (%ID) were calculated from ICP-MS and are normalized for dry weight differences. The AuNP-T cell group exhibited significantly higher gold delivery to the lungs, liver, and bone, while the AuNP group demonstrated higher gold levels within the small intestine. No significant differences were seen in the spleen, kidney, muscle, or brain. An asterisk indicates statistically significant (*P *< 0.05) differences.

We next performed a comprehensive *in vivo *biodistribution study using inductively coupled plasma mass spectrometry (ICP-MS) and ICP-OES to map the location of free PEGylated AuNPs (40-45 nm gold colloid coated with 5000 MW PEG) and AuNPs delivered by T cells. Prior to injection, ICP-OES was performed to determine the absolute gold dose for PEG-AuNPs and AuNP-T cells. Following intravenous injection with PEG-AuNPs or AuNP-T cells, tumors, and organs (bone, brain, heart, intestine, kidney, liver, lungs, muscle, plasma, and spleen) were harvested and analyzed for gold levels using ICP-MS. For PEG-AuNP treated mice, organs were harvested at 4, 8, 24, and 48 h post-injection, while for AuNP-T cell treated mice, organs were harvested at 24 and 48 h (Figure 4a). Predictably, the biodistribution of AuNP-T cells is altered when compared to that of the PEG-AuNPs. As observed in previous studies [13,15], the highest percentages of AuNPs using PEG coating were delivered to the liver and spleen (5.65 and 17.03%, respectively, at 48 h, Figure 4b,c). In comparison, T cells delivered AuNPs to the lung, liver, and spleen, which received 4.76, 33.5, and 2.69% at 48 h, respectively (Figure 4b and 4c). The plasma half-life of the PEG-AuNPs was calculated to be 6.05 h, and no gold was detected in the plasma for the AuNP-T cell group, suggesting no significant AuNP leakage from the T cells during *in vivo *migration. The AuNP-T cell biodistribution over time correlates with the normal biodistribution of human T cells, suggesting that the presence of internalized AuNPs does not significantly change the T cell biodistribution [32]. These data suggest that cellular delivery of AuNP will result in a unique biodistribution pattern that is dependent on the cell type used for delivery.

### T cell delivery increases tumor accumulation of AuNPs

**Figure 5:**
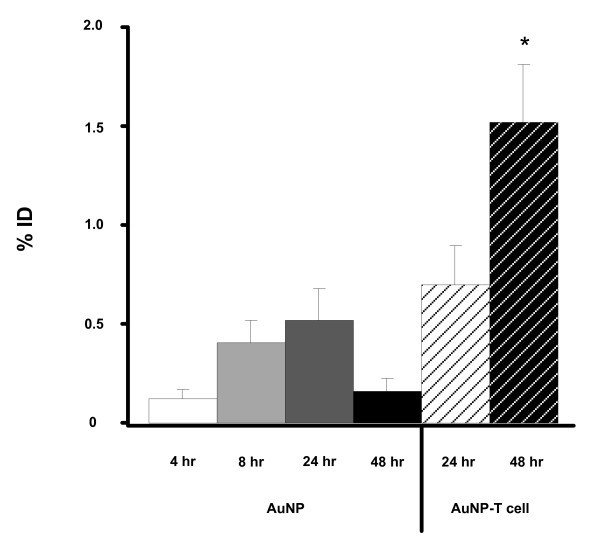
**AuNP-T cells enhance the delivery of gold nanoparticles to the tumor site *in vivo***. Tumor-bearing mice were injected i.v. with PEG-AuNPs or AuNP-T cells. Tumors were subsequently resected at various time points and measured for AuNP content using ICP-MS. Values displayed represent the percentage of injected gold normalized for tumor dry weight differences (mean ± SEM). The percentage of gold delivered by the AuNP-T cells at 48 h represents a significant, four-fold increase over the PEG-AuNP group at 24 h (*P *< 0.01).

Closer examination of LCL tumors following treatment with either PEG-AuNPs or AuNP-T cells showed an increase in AuNP delivery to tumors following cellular transport. For PEG-AuNPs, the highest level of accumulation in tumors was observed at 24 h post-injection, while peak tumor gold accumulation following T cell delivery was seen at 48 h. Using PEG-AuNP, ICP-MS analysis of gold content of excised tumor tissue showed that 0.39 ± 0.33% of ID reached the tumor at 24 h. Whereas, using AuNP-T cells, 1.55 ± 0.72% of the ID localized to the tumor at 48 h (*P *< 0.01) (Figure 5). This represents a four-fold increase in the efficiency of AuNP delivery to the tumor site using T cells as vehicles.

## Discussion

One of the greatest challenges of translating nanotechnologies to the clinical realm is optimizing *in vivo *delivery. Maximizing AuNP accumulation at the tumor site has the potential to enhance photothermal cancer therapy, as well as other applications such as optical imaging. In this study, we show that human T cells can be used to transport AuNPs to distant tumor sites following intravenous administration. Following short term incubation with AuNPs, T cells can be efficiently loaded with over 14,000 AuNPs per cell without affecting cell viability, proliferation, and cytokine production. Importantly, T cells loaded with AuNPs retain their ability to migrate *in vitro*, and demonstrate tumor-specific homing in mice. Using T cells as a vehicle to deliver AuNPs resulted in a four-fold increase in the efficiency of AuNP tumor accumulation, demonstrating that active transport of AuNPs by cellular chaperones is superior to that of passive accumulation through the EPR effect.

Stephan et al. [33] recently demonstrated that synthetic drug-carrier nanoparticles could be stably conjugated to the surface of immune cells, including T cells, for delivery of therapeutic molecules. In these studies, T cells efficiently carried surface-tethered nanoparticles to tumors in mice, and when loaded with cytokines to support T cell growth, dramatically increased antitumor efficacy. However, our study conclusively demonstrates *in vivo *that internal loading of AuNPs in T cells can improve tumor localization, and thus may be a useful technology for a variety of nanoparticle based therapies.

In this study, we elect to use AuNPs. AuNPs are known to have low cytotoxicity, and gold has been used in humans for the treatment of arthritis for over 50 years [34], which makes AuNPs a logical choice in the pursuit of clinical applications. For this study, 40-45 nm gold colloidal nanospheres were selected for internalization by activated human T cells. The internalization of nanoparticles by cells is believed to be accomplished predominantly by receptor-mediated endocytosis, and particle size is an important variable in determining the kinetics of cellular uptake, with maximal uptake in a size range of 40-50 nm [35,36]. We selected the size of our AuNPs for this proof-of-concept delivery study to optimize nanoparticle cellular uptake. We modulated the degree of nanoparticle internalization by altering the concentration of nanoparticles incubated with the T cells (Figure 1c). We also evaluated nanoparticle uptake using T cells isolated from three different human donors (Figure 1c) and saw only small variation, suggesting that this technique could be extrapolated to the T cells of any patient.

The internalized gold colloid used in this study also had no detrimental impact on the viability or function of activated human T cells in vitro (Figure 2), and the T cells were able to migrate to tumors *in vivo *while maintaining their AuNP payload (Figure 3). In addition to their ability to carry AuNPs to tumors, T cells can be selected for tumor-specificity for adoptive immunotherapy studies [37-39]. Furthermore, T cells may be genetically engineered to improve their function [40,41] or enhance their ability to migrate to tumors *in vivo*[42,43]. It has been demonstrated that systemically administered AuNPs tend to accumulate mainly in the perivascular regions of the tumor [11], limiting passive accumulation of nanoparticles by the EPR effect to well-vascularized regions of the tumor. T cells may naturally localize to tumors, and tumor-specific T cell clones have been demonstrated to penetrate into the hypoxic cores of the tumors *in vivo *[44]. The more extensive infiltration of tumor sites by antigen-specific T cells may permit enhanced penetration of the tumor when compared to freely-injected nanoparticles, potentially augmenting therapeutic efficacy.

The use of T cell vehicles also significantly affects nanoparticle biodistribution (Figure 4). Freely injected nanoparticles (40-45 nm gold colloidal nanospheres coated with 5000 MW PEG) accumulate most significantly in well-vascularized organs such as the liver, spleen, kidney, and gut (Figure 4). Maximal AuNP tumor accumulation for the freely injected PEG-AuNP group is seen at 24 h (Figure 5). After 24 h, increased gold content for the PEG-AuNP group is seen in the spleen, liver, and kidney with a corresponding decrease in gold content within the tumor and other organs, which represents a shift towards AuNP clearance.

AuNP-T cells present a much different biodistribution from the systemically administered nanoparticles that correlates with the expected biodistribution of T cells. After adoptive transfer of AuNP-T cells, a large percentage of the ID is seen within the liver and lungs at 24 h. T cells are known to accumulate within the liver and lungs after administration due to the vascularity and number of adhesion molecules present in these organs [45]. This pattern of T cell migration is consistent with the biodistribution of adoptively transferred T cells seen in previous studies [33,45]. AuNP-T cells are also seen accumulating in the spleen and bone of the mice; these locations are also normal reservoirs of T cells [46]. The large number of AuNP-T cells seen in the liver likely represents apoptotic T cells. This large accumulation is not observed by bioluminescence imaging in Figure 3, and the liver is a known site where apoptotic T cells are entrapped [47]. Tumor accumulation of AuNP-T cells increases from 24 to 48 h as T cells escape from the lungs and migrate to the tumor (Figure 5). The biodistribution of AuNP-T cells matches the expected biodistribution of normal activated T cells, suggesting that AuNP biodistribution can be modulated based on the selection of the cellular vehicle. In the case of T cells, it is possible that the biodistribution may be altered to to further favor tumor accumulation and persistence by manipulating cell culture conditions [45] or by genetic modification of T cells [43].

Using T cells as cellular vehicles for AuNP delivery, we achieve a four-fold increase in tumor delivery efficiency at 48 h when compared to freely injected PEG-coated AuNPs at 24 h (Figure 5). This represents a significant increase in delivery efficiency (*P *< 0.01, Student's *t*-test) using T cells. These results demonstrate for the first time that T cells can be used to enhance AuNP delivery to a tumor *in vivo*. The use of AuNPs and T cells together combines the photothermal therapy and imaging advantages of AuNPs with the immunotherapy and biodistribution advantages of T cells. Future directions will focus on utilizing the AuNP-T cell system for cancer therapy by modifying the T cells to further enrich AuNP tumor accumulation and enhance anti-tumor effects.

## Conclusions

In this study we demonstrate the internalization of AuNPs into activated human T cells for the delivery of nanoparticles *in vivo*. AuNP uptake has no negative impact on T cell viability, proliferation, or immune function, and T cells are able to transport the AuNP payload to tumor sites *in vivo*. Furthermore, the use of T cells as a AuNP vehicle enhances *in vivo *delivery efficiency by four-fold. This delivery method alters the biodistribution of gold compared to freely injected AuNPs, and demonstrates that the selection of a particular cellular vehicle may dictate AuNP biodistribution.

## Methods

### AuNP synthesis and PEGylation

Gold(III) chloride trihydrate (HAuCl_4 _3H_2_O 99%) and potassium carbonate anhydrous (K_2_CO_3 _99%) were purchased from Sigma-Aldrich (St. Louis, MO). Deionized water was provided by a Milli-Q system. In this synthesis method, Au^3+ ^is reduced to Au^0 ^using CO as a reducing agent. A 0.38 mM HAuCl_4 _solution was prepared and aged in an amber bottle at 4°C for a minimum of 72 h prior to use. After aging the chloroauric acid solution, the temperature was allowed to gradually rise to 16°C. A 1.8 mM K_2_CO_3 _solution was then prepared by adding 75 mg of K_2_CO_3 _to the aged 200 mL HAuCl_4 _solution. This solution was aged for 30 min prior to aeration with CO gas. A 40 mL volume of the aged solution was added to the beaker and stirred continuously prior to aeration. CO gas (Matheson-Trigas) was injected into the continuously stirring solution at a flow rate of 30.5 mL/min. The CO flow was controlled via a flow rate control valve. A visible color change from clear to dark purple to red is observed during synthesis, indicating formation of AuNPs. TEM images were taken to confirm size and monodispersity. Particles were sterilized by filtration through a 0.22 μm polyethersulfone filter. To stabilize the particles in preparation for mouse injection, 0.5 mM polyethylene glycol-thiol (PEG-SH, MW = 5 kD, Nektar) was added to the particles. After a 24-h incubation, excess PEG-SH was removed by centrifugation and PEGylated particle stability was confirmed by increasing solution tonicity with 1 M NaCl. Dynamic light scattering measurements were taken to assess the hydrodynamic diameter of the PEGylated gold colloid.

### T cell isolation and preparation

Peripheral blood was obtained with informed consent from willing healthy donors using a Baylor College of Medicine Institutional Review Board approved protocol. Peripheral blood mononuclear cells (PBMC) were isolated by Ficoll gradient centrifugation (Lymphoprep, Nycomed, Oslow, Norway). PBMC were used to generate EBV-transformed B cells lines (LCL) and T cell lines. LCL and T cells were maintained in RPMI 1640 supplemented with 10% fetal calf serum (FCS; Hyclone, Logan, UT) and 2 mM GlutaMAX (Invitrogen, Carlsbad, CA). For T cell expansion, non-tissue culture treated 24-well plates were coated with OKT3 (1 μg/mL; Ortho Pharmaceuticals, Raritan, NJ) and anti-CD28 antibody (1 μg/mL; BD Biosciences, San Diego, CA) overnight at 4°C. Plates were washed and 2 × 10^6 ^PBMC were plated per well in complete RPMI supplemented with 100 U/mL recombinant human interleukin-2 (IL-2). On day 3, T cell blasts were harvested and further expanded or transduced in IL-2 supplemented media.

### T cell internalization of AuNPs

Day 7 OKT3 blasts were harvested and suspended in complete RPMI supplemented with IL-2 and 0, 0.05, 0.1, 0.25, 0.5, or 1 nM of AuNPs for 24 h (1 mole = 6.022 × 10^23 ^nanoparticles). Cells were harvested and washed a minimum of three times using 1 × phosphate-buffered saline (PBS) prior to subsequent experiments. To confirm loading, T cells were imaged using darkfield microscopy. To quantitatively characterize loading, 2 × 10^6 ^T cells per sample were prepared for ICP-OES analysis by digesting the cells in three parts trace metal grade hydrochloric acid (Fisher Scientific, Pittsburgh, PA) and one part trace metal grade nitric acid (EMD Chemicals, Gibbstown, NJ) overnight. Samples were then diluted to 10 mL in distilled water and filtered. T cells incubated with media alone were used as a control.

### T cell viability and functionality after AuNP loading

To determine the effect of AuNP loading on T cell phenotype, we used the following monoclonal antibodies conjugated to FITC, PE, PerCP, or APC (BD Biosciences): CD3, CD4, CD8, CD45RA, CD45RO, CD56, CD62L, CCR5, and CCR7. An Annexin V apoptosis detection kit (BD Biosciences) was used to determine T cell viability post-AuNP loading. Cells were analyzed using a FACSCalibur flow cytometer (BD Biosciences) and FCSExpress software (De Novo Software, Los Angeles, CA). A [^3^H] thymidine incorporation assay was used to assess the effects of AuNP loading on T cell proliferation. Following AuNP loading, T cells were seeded in triplicate into 96-well round bottom plates at 1 × 10^5 ^cells per well in complete RPMI containing 100 U/mL IL-2 for 24 h. T cells were then pulsed with 5 μCi [^3^H] thymidine (Amersham Pharmacia Biotech, Piscataway, NJ) overnight. Cells were then harvested onto glass filter strips and analyzed using a TriCarb 2500 RT β-counter (Packard Biosciences, Downers Grove, IL). To determine if AuNP-loaded T cells retain the ability to migrate *in vitro*, we used a transwell migration assay. T cells were labeled with 50 μCi Chromium^51 ^(Cr^51^; MP Biomedicals, Solon, OH) and 1.5 × 10^5 ^cells were placed in the upper chamber of 24-well 6.5 mm diameter, 5 μm pore size transwell chambers (Costar Transwell, Corning, NY). Media alone or LCL tumor supernatant was placed in the bottom chamber. Plates were then incubated for 3 h at 37°C. Cells in the bottom chamber were then harvested and analyzed using a γ-counter (Cobra Quantum, Perkin Elmer, Shelton, CT). Specific migration was calculated using the following equation: Specific Migration (%) = (Experimental [LCL supernatant] - Spontaneous [media alone])/(Maximum [1.5 × 10^5 ^cells] - Spontaneous [media alone]) × 100. To measure the ability of AuNP-loaded T cells to secrete IFN-γ following mitogenic stimulation, 2 × 10^5 ^T cells were seeded into 96-well round bottom plates for 24 h. T cells were then stimulated with 25 ng/mL phorbol myristate acetate (PMA; Sigma-Aldrich, St. Louis, MO) and 1 μg/mL Ionomycin (I; Sigma-Aldrich). Following 2 h of PMA-I stimulation, Brefeldin A (Sigma) was added to allow for intracellular cytokine retention. Four hours later, cells were permeabilized using 1% Saponin (Sigma) and IFN-γ expression was detected by intracellular cytokine staining using PE-conjugated anti-IFN-γ monoclonal antibody (BD Biosciences).

### *In vivo *delivery studies

#### SCID xenograft model

*In vivo *migration, AuNP delivery, and biodistribution studies were performed using severe combined immune deficient mice (SCID [strain ICR-Prkdc(scid)]; Taconic, Hudson, NY). All mouse experiments were performed under a Baylor College of Medicine Institutional Animal Care and Use Committee (IACUC) approved protocol. 1 × 10^7 ^LCL tumor cells were resuspended in Matrigel (BD Biosciences) and injected subcutaneously (s.c.) into the shaved right flanks of mice. Tumors were allowed to establish and grow to at least 0.5 mm × 0.5 mm in size (2-3 weeks) before use.

#### Mouse injections and sample collection

To prepare AuNP-T cell injections, T cells were prepared as above and incubated with 0.5 nM AuNPs for 24 h. Cells were harvested and washed extensively using 1 × PBS prior to injection. For delivery studies, mice received either PBS, 1 × 10^7 ^AuNP-T cells, or 1 × 10^11 ^PEGylated AuNPs via the tail vein in a 200 μL bolus. These dosages were selected based on previous *in vivo *studies using AuNPs and adoptively transferred T cells. To determine optimal time points for delivery analysis, tumors were resected at either 4, 8, 24, or 48 h for the PEGylated AuNP group and 24 or 48 h for the AuNP-T cell group. In addition, plasma as well as portions of the liver, spleen, kidneys, small intestine, muscle, heart, lung, bone, and brain were also collected for analysis at 4, 8, or 24 h for the PEGylated AuNP group and 24 or 48 h for the AuNP-T cell group. All tissues, including tumors, were flash frozen with liquid nitrogen after collection and stored at -80°C until analysis.

#### Bioluminescent imaging

To determine if AuNP-loaded T cells can migrate to tumors *in vivo *and thereby deliver AuNP to the tumor site, T cells were transduced with retrovirus encoding GFP*luc *as previously described by our group [48]. Transduced cells were then loaded with AuNPs for 24 h then injected intravenously (i.v.) via the tail vein (1 × 10^7 ^T cells per mouse). Forty-eight hours post-T cell infusion, the biodistribution of T cells was visualized using the *In Vivo *Imaging System (IVIS; Xenogen) following intraperitoneal (i.p.) injection of 150 mg/kg D-luciferin (Xenogen, Alameda, CA).

#### Ex vivo tissue analysis and imaging

To image AuNP-T cells within the tumor, tumors were thawed in a 37°C water bath and embedded in optimal cutting temperature (O.C.T.) compound (Sakura Finetek USA, Inc., Torrence, CA) using dry ice. The embedded tissue was then sectioned into 8 μm slices using a cryostat, dried overnight at room temperature, and stored at -80°C. Tissue sections were then fixed with acetone and stained for CD3 using anti-CD3 (Abcam ab5690, Cambridge, MA) as the primary antibody and the Invitrogen Histostain^® ^Plus Broad Spectrum (AEC) kit. Slides were coverslipped with immunomount (Thermo Scientific, Pittsburgh, PA) and imaged by bright field and dark field microscopy. Resected mouse tissues were prepared and analyzed for gold content using ICP-MS and ICP-OES. Samples were lyophilized and weighed, then digested and prepared as previously described. Samples of the AuNP-T cell and AuNP boluses were also analyzed to confirm the amount of gold systemically administered.

### Statistical methods

For the biodistribution analysis, we performed a one-way ANOVA followed by Tukey's method. Each organ was examined individually at various time points for each treatment group (*n *= 3 for all time points and treatment groups). Significance was set at *P *< 0.05. An asterisk indicates significant differences between every possible AuNP:AuNP-T cell comparison pair at all time points for each organ. For the tumor delivery analysis, we used a Student's *t*-test to compare AuNP systemic administration to AuNP-T cell delivery. For this study, *n *= 8 for the AuNP group, while *n *= 11 for the AuNP-T cell group.

## Abbreviations

AuNPs: gold nanoparticles; EPR: enhanced permeability and retention; FCS: fetal calf serum; ICP-MS: inductively coupled plasma mass spectrometry; ICP-OES: inductively coupled plasma optical emission spectrometry; ID: injected dose; i.p., intraperitoneal; i.v., intravenous; NIR: near infrared; O.C.T.: optimal cutting temperature; PBMC: peripheral blood mononuclear cells; PMA: phorbol myristate acetate; PBS: phosphate-buffered saline; PTT: photothermal therapy; PEG: polyethylene glycol; s.c., subcutaneous; TEM: transmission electron microscopy.

## Competing interests

The authors declare that they have no competing interests.

## Authors' contributions

LK and AB performed the *in vitro *and *in vivo *characterizations of the AuNPs, the mouse studies, the tumor histology, the ICP-MS and ICP-OES analyses, and drafted the manuscript. JY synthesized and characterized the AuNPs. NL assisted with the ICP-OES and ICP-MS analyses. JK assisted with the tumor histology and ICP-MS and ICP-OES analyses. AF and RD participated in the design and coordination of the study, as well as assisted in the drafting of the manuscript.

## Supplementary Material

Additional file 1 **Figure S1 Gold colloid size distribution**. Particle sizes were determined using TEM. A total of 585 particles were examined over multiple images to generate the histogram. **Figure S2 AuNP-loading does not affect T cell phenotype**. T cells were loaded in the presence of 0.5 nM AuNP for 24 h and subsequently stained with a panel of antibodies and analyzed by flow cytometry. Click here for file
