# Supplementary figures and images for: T cells enhance gold nanoparticle delivery to tumors in vivo
Source: Nanoscale Res Lett. 2011 Apr 4;6(1):283. doi: 10.1186/1556-276X-6-283 (PMC3211348; doi:10.1186/1556-276X-6-283)

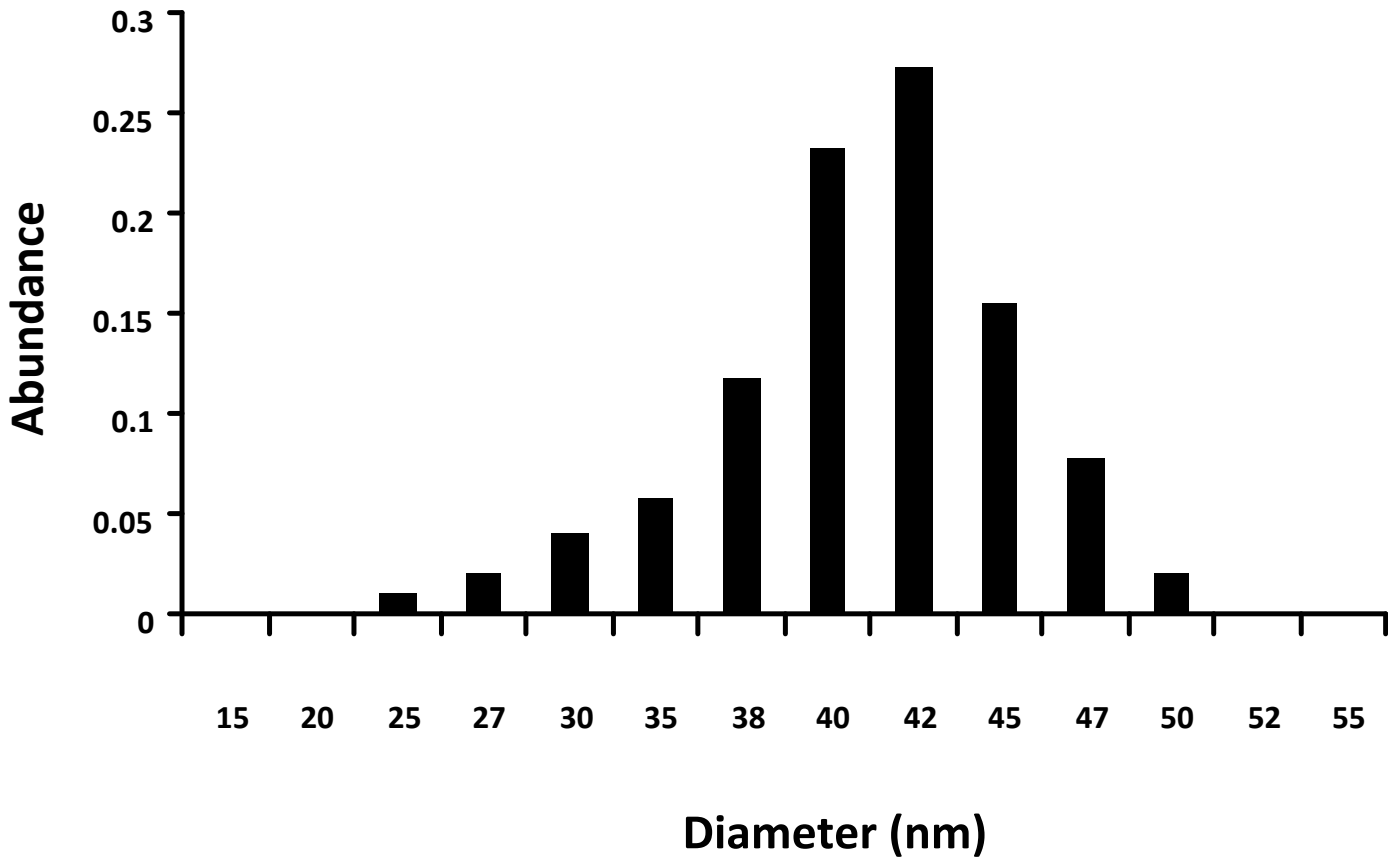

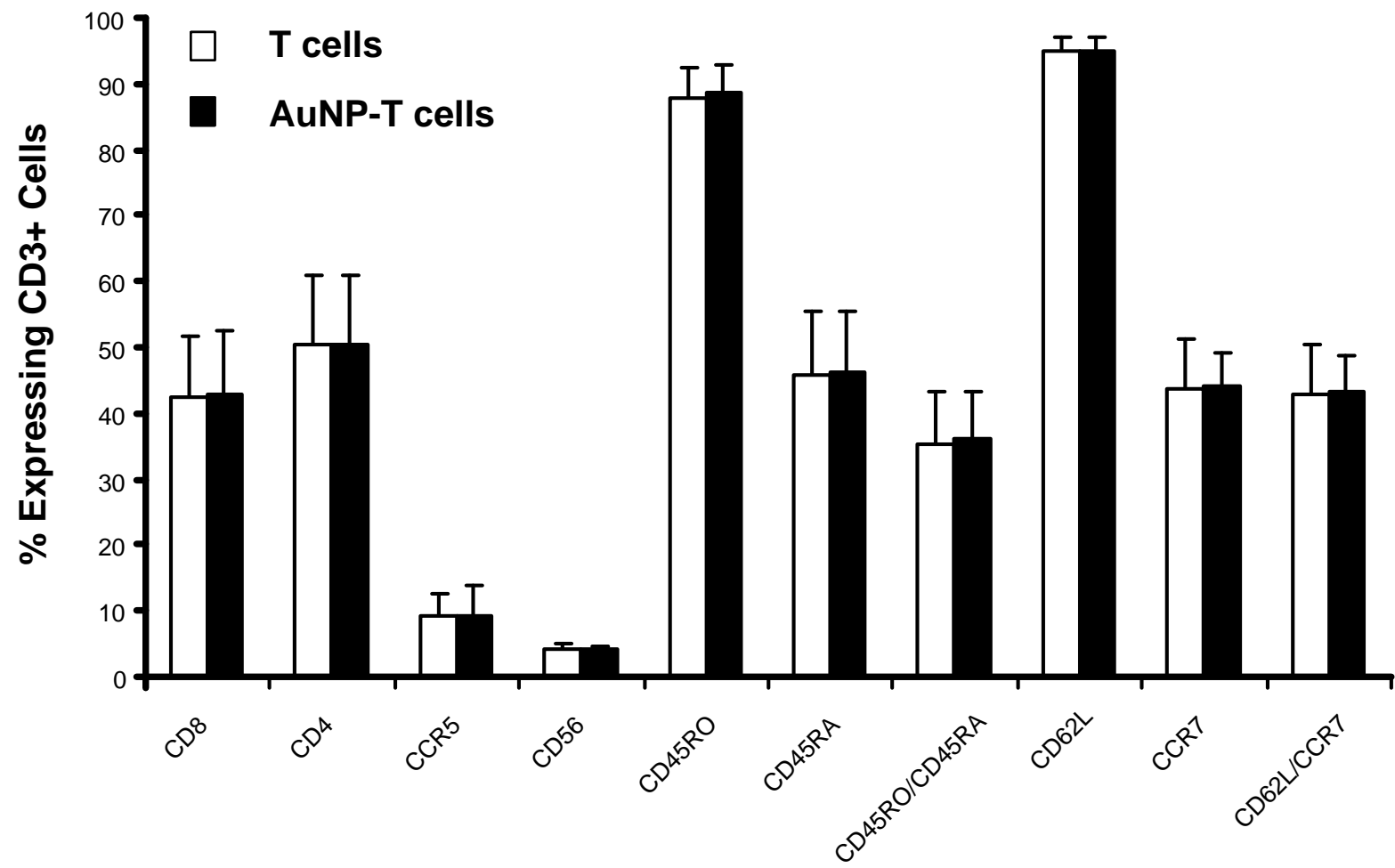

Supplement: Additional file 1 — Figure S1 Gold colloid size distribution. Particle sizes were determined using TEM. A total of 585 particles were examined over multiple images to generate the histogram. Figure S2 AuNP-loading does not affect T cell phenotype. T cells were loaded in the presence of 0.5 nM AuNP for 24 h and subsequently stained with a panel of antibodies and analyzed by flow cytometry. [file 1556-276X-6-283-S1.PDF]
